# Case report: Hepatotoxicity and nephrotoxicity induced by methotrexate in a paediatric patient, what is the role of precision medicine in 2023?

**DOI:** 10.3389/fphar.2023.1130548

**Published:** 2023-05-02

**Authors:** Ali El Rida El Masri, Caroline Tobler, Breunis Willemijn, Andre O. Von Bueren, Marc Ansari, Caroline Flora Samer

**Affiliations:** ^1^ Division of Clinical Pharmacology and Toxicology, Department of Anaesthesiology, Pharmacology, Intensive Care and Emergency Medicine, Geneva University Hospitals, Geneva, Switzerland; ^2^ Division of Pediatric Oncology and Hematology, Department of Women, Child and Adolescent, University Geneva Hospitals, Geneva, Switzerland; ^3^ Cansearch Research Platform for Pediatric Oncology and Hematology, Department of Pediatrics, Gynecology and Obstetrics, Faculty of Medicine, University of Geneva, Geneva, Switzerland; ^4^ Department of Oncology and Children’s Research Center, Faculty of Medicine, University of Zurich, Zurich, Switzerland; ^5^ Department of Clinical Pharmacology and Toxicology, Faculty of Medicine, University of Geneva, Geneva, Switzerland; ^6^ Department of Anaesthesiology, Pharmacology, Intensive Care and Emergency Medicine, Faculty of Medicine, University of Geneva, Geneva, Switzerland

**Keywords:** hepatotoxicity, pharmacokinetic and pharmacodynamic parameters, nephrotoxicity, pharmacogenomic, pediatric case report

## Abstract

Methotrexate is an immunosuppressant and chemotherapeutic agent used in the treatment of a range of autoimmune disorders and cancers. Its main serious adverse effects, bone marrow suppression and gastrointestinal complications, arise from its antimetabolite effect. Nevertheless, hepatotoxicity and nephrotoxicity are two widely described adverse effects of methotrexate. Its hepatotoxicity has been studied mainly in the low-dose, chronic setting, where patients are at risk of fibrosis/cirrhosis. Studies of acute hepatoxicity of high dose methotrexate, such as during chemotherapy, are scarce. We present the case of a 14-year-old patient who received high-dose methotrexate and subsequently developed acute fulminant liver failure and acute kidney injury. Genotyping of *MTHFR* (Methylene tetrahydrofolate reductase gene), *ABCB1* (codes for P-glycoprotein, intestinal transport and biliary excretion), *ABCG2* (codes for BCRP, intestinal transporter and renal excretion) and *SLCO1B1* (codes for OATP1B1, hepatic transporter) identified variants in all the genes analysed that predicted a reduced rate of methotrexate elimination and thus may have contributed to the clinical situation of the patient. Precision medicine involving pharmacogenomic testing could potentially avoid such adverse drug effects.

## Introduction

Methotrexate, a competitive inhibitor of dihydrofolate reductase, is used for the treatment of an array of auto-immune disorders and cancers (osteosarcoma, acute lymphoblastic leukaemia, Hodgkin’s lymphoma). It enters cells via different transporters according to the cell type, notably OATP1B1 (Mikkelsen et al., 2011), an organo-anion transporter that mainly transports organic anions across cell membranes in hepatocytes (encoded for by the *SLCO1B1* gene). Although fecal excretion of methotrexate is minor, reduced OATP1B1 activity increases methotrexate plasma concentration, suggesting that methotrexate is characterized by a highly efficient enterohepatic circulation. Reduced OATP1B1 activity simultaneously reduces the liver uptake of methotrexate and increases its plasma concentration (van de Steeg et al., 2013). An association between SLCO1B1 mutations and methotrexate clearance/toxicity is now established in clinical practice (Treviño et al., 2009; Lopez-Lopez et al., 2011; Mikkelsen et al., 2011; Ramsey et al., 2012; Ramsey et al., 2013; van de Steeg et al., 2013). When bound to methotrexate, dihydrofolate reductase (DHFR) no longer converts dihydrofolate (DHF) to tetrahydrofolate (THF) (Howard et al., 2016). DHF and THF are essential for the formation of thymidine and purines, which are needed for the synthesis of DNA (Howard et al., 2016; Swissmedic). Methotrexate also interacts with other intracellular enzymes involved in the folate cycle, particularly 5,10-methylenetetrahydrofolate reductase (MTHFR). Eventually, methotrexate is eliminated by ABC transporters, mainly Breast Cancer Resistance Protein (BCRP) and P-glycoprotein (P-gp) (Mikkelsen et al., 2011). BCRP transports polyglutamate derivatives of methotrexate as well as methotrexate, and therefore a reduced BCRP functional capacity could lead to higher concentrations of intracellular polyglutamate derivatives of methotrexate and thus increase its toxicity (Volk and Schneider, 2003).

Folates and antifolates are mostly bivalent anions, meaning they diffuse poorly into the tissues and are actively transported between different organ systems using many transporters. Monoglutamate derivatives of folate are actively absorbed from the intestine using the proton coupled folate transporter (PCFT) which is expressed by different cell lineages but requires an acidic environment to function. In the liver, they are either converted to polyglutamate derivatives via folylpolyglutamate synthetase (FPGS) or eliminated in the bile. When needed, they are converted back to monoglutamate folate derivatives and transported in the systemic circulation and into normal cells via the reduced folate transported (RFC). Folates and antifolates are both substrates of RFC and PCFT, transporters which function bidirectionally. However, their efflux capacity is suppressed by organic phosphates and a neutral intracellular pH respectively. This can explain, at least partially, why their dysfunctional states are not implicated in antifolate toxicity. Folate receptors are another way into cells for folates/antifolates, however, with a much lower affinity to antifolates, this is less relevant to the case of an antifolate toxicity (Zhao et al., 2011; Visentin et al., 2012).

The most common adverse drug reactions associated with the use of high-dose methotrexate, defined as a dose greater than 500 mg/m^2^, are mucositis, myelosuppression, gastrointestinal toxicity, neurological toxicity, and less commonly hepatotoxicity, for which no preventative measures can be taken. Acute renal failure, a less common adverse drug reaction, is seen with high-dose methotrexate, caused by precipitation of the methotrexate in the renal tubules (Howard et al., 2016). Chemotherapy protocols include preventive measures to avoid this complication, such as a regular monitoring of methotrexate plasma levels, hyperhydration to allow alkalinisation of the urine, and administration of leucovorin (folinate) according to the plasma levels of methotrexate as well as administration of the antidote (carboxypeptidase) (Howard et al., 2016; Boelens et al., 2018).

Methotrexate-induced toxicity varies greatly between individuals, even with similar doses and timelines of administration. Several studies (Lopez-Lopez et al., 2011; D’Cruz et al., 2020; Zhang et al., 2022; Erdilyi et al., 2008) have investigated a possible link between genetic variability of different transporters/enzymes and methotrexate toxicity, potentially explaining its toxicity by the inter-individual variability in methotrexate pharmacokinetic parameters. Various genetic polymorphisms are thought to have an impact on the clearance of methotrexate and thus its toxicity. To date, multiple variants of enzymes and transporters involved in the elimination of methotrexate have been identified that may reduce their activity.

Here we describe the case of a paediatric patient who developed fulminant toxic hepatitis and acute kidney injury following a single high dose of methotrexate. Pharmacogenetic testing revealed reduced activity of the P-gp, BCRP and OATP1B1 transporters as well as the MTHFR enzyme, predisposing the patient to develop toxicities associated with reduced methotrexate clearance.

## Case

**GRAPH 1:**
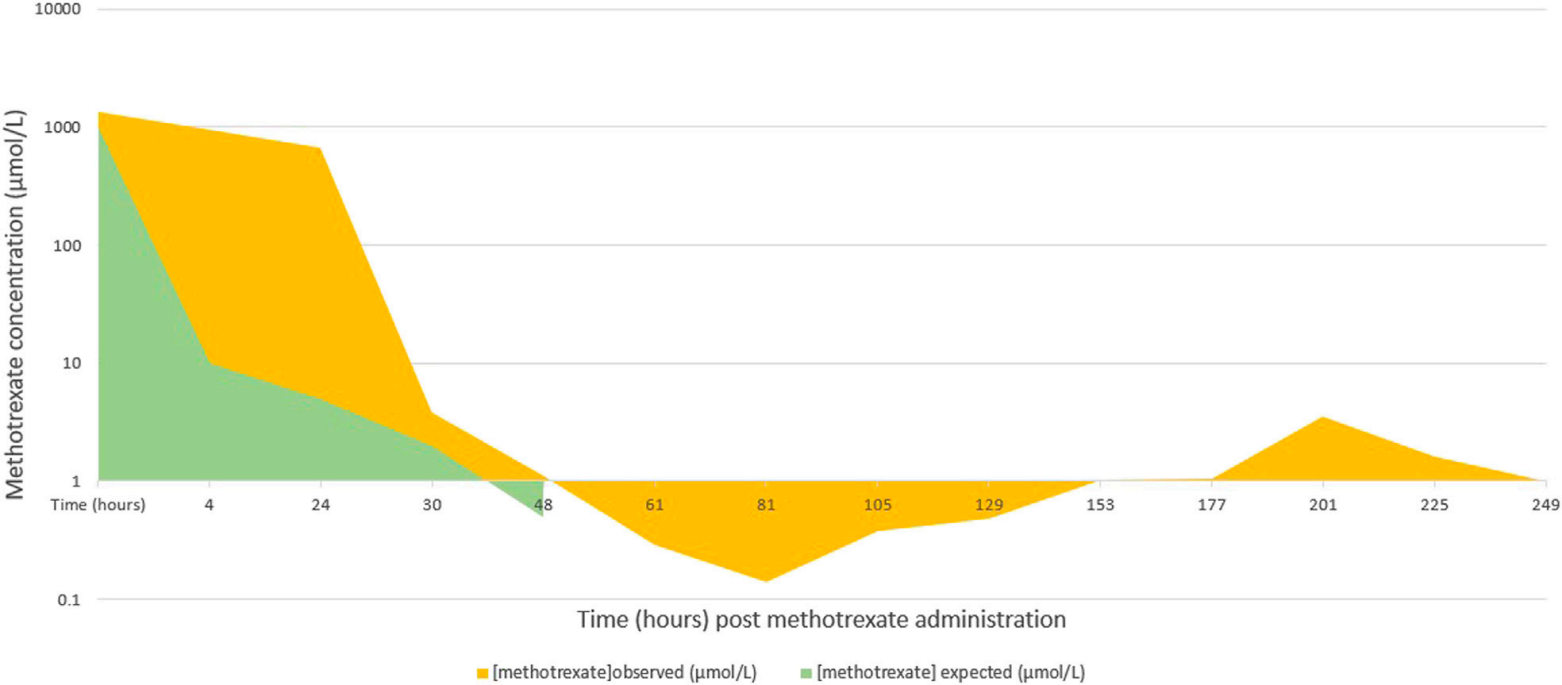
Evolution of the concentrations of methotrexate over 11 days.

A 14-year-old female patient with a high-grade osteosarcoma of the right tibia was treated according to the EURASMOS protocol. On day 23 of the protocol, a first dose of intravenous methotrexate (12 g/m^2^) was administered. Prior to administration of the high dose methotrexate (HDMTX), the patient’s renal function was in the normal range (creatininemia 46 umol/L, GFR 130 ml/min/1.73 m^2^ (van de Steeg et al., 2013) according to the Schwartz equation. Liver enzymes were slightly elevated (AST 25 U/L (N < 20 U/L), ALT 31 U/L (N < 22 U/L) with no sign of cholestasis (total bilirubin 4 umol/L). Synthetic liver function was not tested at this stage. Prior to administration of the high dose methotrexate, the patient received hyperhydration as per protocol (200 ml/m^2^/h with 40 mEq/L sodium bicarbonate for 6 h prior to HDMTX). Hyperhydration was continued (125 ml/m^2^/h) during the methotrexate administration. Methotrexate plasma levels measured (using LC-MS) at H4 and H24 were 1,356 and 937 μmol/L(Graph 1), respectively [expected methotrexate plasma levels at H24: <10 μmol/L (Pignon et al., 1994; Holmboe et al., 2012)]. At around 24 h, the patient began to show altered state of consciousness and haemodynamic instability (treated with noradrenaline and adrenaline) and was intubated (propofol, rocuronium, midazolam and fentanyl were administrated). As a prevention for infection, meropenem was also administered.

The laboratory showed severe renal failure with an increased creatininemia to 268 μmol/L and hyperkaliemia up to 6.1 mmol/L. She also suffered from severe hepatic impairment with a significant elevation of the liver enzymes (AST 10,121 U/L, ALT > 6,000 U/L), cholestasis (total bilirubin 39 μmol/L) and an impaired hepatic synthetic function (factor V 6.9%, Quick 29%, INR 2.28).

Suspecting acute renal failure secondary to methotrexate use, the patient received leucovorin (100 mg/m^2^ QDS) for the competitive inhibition of the activity of methotrexate, glucarpidase (50 U/kg) to eliminate methotrexate faster, cholestyramine to decrease enterohepatic circulation and N-acetylcysteine to reduce the oxidative damage on the liver. Hyperhydration was increased up to 4,500 ml/m^2^/24 h to maintain urine alkalinisation.

Nevertheless, presenting with a severe refractory metabolic acidosis and hyperkalaemia, haemodialysis was initiated and a transfer to the intensive care unit of the University Hospital of Geneva was organised for liver organ support and liver dialysis.

The doses of methotrexate administered were controlled several times by the pharmacy, and the patient was not taking any other medications that could interact with methotrexate.

Methotrexate plasma levels were measured regularly from H0 to H273. The maximum level was measured at H4 (1,356 μmol/L). After 8 days of renal dialysis and 11 days of hepatic dialysis (Molecular adsorbent recirculating system (MARS), an extracorporeal hepatic support system that allows dialysis and ultrafiltration), renal and hepatic function improved significantly. There was a rebound in the methotrexate levels noted after a couple of sessions of haemodialysis which can be attributed to the redistribution of methotrexate from other compartments not initially accessible for removal. Therefore, multiple sessions of high flux haemodialysis are usually necessary in similar cases (Bleyer, 1977).

Regarding the patient’s future chemotherapy, it was decided not to re-administer high-dose methotrexate. The methotrexate blocks were replaced by a course of ifosfamide/etoposide and carboplatin/etoposide. Given that the changes in the treatment plan were made very recently, we do not as of yet have any information on the treatment outcome.

## Results of the genotyping of the germline DNA

## Discussion

High-dose methotrexate is commonly used as an anticancer agent in the paediatric population, and although the most common toxicities are reversible, the mortality and morbidity associated with such a therapy remains important. Discontinuation of treatment due to an adverse effect is also associated with a poorer cancer prognosis (Song et al., 2021).

In our case, the patient developed severe renal and hepatic failure secondary to a high dose of methotrexate. Pharmacogenetic testing was subsequently performed and showed multiple polymorphisms increasing her risk of developing adverse effects (Table 1). Pre-emptive genotyping could have prevented the adverse effects, either with an unguided dose adjustment, a pre-emptive renal protection therapy, or with the use of an alternative chemotherapeutic agent. In the absence of guidelines for adjusting the dose of methotrexate according to the genetic profile, the use of different therapies as first line agents could be considered.

**TABLE 1:** The pharmacogenetics panel revealed mutations of the 4 pharmacogenes *ABCB1*, *MTHFR*, *ABCG2* and *SLCO1B1* that predict a reduced activity of P-gp, MTHFR, BCRP, and OATP1B1 respectively.

Gene	Variants detected	Genotype*	Interpretation/predicted phenotype
*ABCB1* (P-gp)	c.3435C>T	T/T	Reduced activity
	c.1236T>C	T/C	
	c.2677G>T	G/T	
	c.210A>G	A/G	
*MTHFR*	c.1298A>C	A/C	Reduced activity
	c.677C>T	C/T	
*ABCG2*	c.421C>A	C/A	Reduced activity
*SLCO1B1*	c.388A>G	G/G	Reduced activity
	c.521T>C	T/C (*1/*15 or *1/*17)	

In two pharmacokinetic studies of patients who received high dose methotrexate (mean dose around 13.2 and 12 g/m^2^, similar to our patient’s), the mean plasma concentration of methotrexate was well below 100 μmol/L after 24 h of the infusion and below 10 μmol/L at 48 h (Pignon et al., 1994; Holmboe et al., 2012). Our patient had more than 10-fold increase in the plasma level of methotrexate at 24 h post-infusion, which could be explained by the genetic polymorphisms detected. The rescue therapy provided had quickly rectified the levels, with a 48-h level similar to that of other patients in both studies.

The most important physiological factor impacting methotrexate elimination is renal function. It is directly affected by the level of hydration, as volume depletion due to diarrhea and vomiting results in renal hypoperfusion. This creates a vicious cycle between methotrexate accumulation in renal tubules and direct toxic damage to the renal tubules, from prolonged contact, which in turn further worsens renal function (Perazella and Moeckel, 2010).

The elimination of methotrexate has been subject of various studies. The current consensus is that it involves different pathways, including P-gp, MTHFR, OATP1B1 and BCRP. The frequency of the polymorphisms associated with a reduced metabolism/elimination of their substrates varies according to the population studied and the genetic panel analysed. Given the degree of heterogeneity of the study designs looking into the frequency of polymorphisms, the frequency of the variants vary. For example, SLCO1B1*15 frequency was estimated between 3% in African/Oceanian population to around 15% in the European population and 24% in the American one (Oshiro et al., 2010).

The P-gp (encoded by the ABCB1 gene) is located in several tissues, such as the intestines, kidneys, liver, immune system, blood-brain barrier and placenta. Its role is to expel drug substrates (Hodges et al., 2011; PharmGKB, 2023). In a physiological setting, it removes methotrexate from the intestinal/renal/hepatic cells into the gut/urine/bile respectively. Polymorphisms of *ABCB1* have been described, the main ones being associated with reduced transport (Hodges et al., 2011). In general, available studies indicate that patients with certain allelic variants (3435TT and/or 2677 T or A genotype) require lower doses of opioids, which are P-gp substrates, and at greater risk of central adverse effects such as drowsiness and confusion (Ross et al., 2008; Sadhasivam and Chidambaran, 2012; Hajj et al., 2013; Rhodin et al., 2013). These polymorphisms may potentiate the penetration of active substrates in the cells and increase the risk of toxicity. The MTHFR enzyme plays a key role in folate metabolism and is involved in the pharmacodynamics of several drugs. Data from the literature report that the c.677C>T variant is associated with an increased risk of methotrexate toxicity via a decreased MTHFR enzyme activity (Yang et al., 2012). The OATP1B1 transporter is involved in the intracellular influx of endogenous and exogenous compounds. This protein is expressed mainly on the basolateral membrane of hepatocytes (Oshiro et al., 2010). The OATP1B1 gene is polymorphic, the most described variant being c.521T>C (rs4149056), for which the minor C allele is associated with reduced transport *in vitro* and reduced elimination of certain drugs *in vivo* (Ramsey et al., 2014). BCRP, an ABC family transporter, is involved in the transport of various xenobiotics, including MTX (Breedveld et al., 2004; Yu et al., 2021). It is most highly expressed in the brain, small intestine, cervix and uterus (Fohner et al., 2017). With the presence of reduced BCRP activity, less methotrexate is expelled into the small intestine. The two most common and well-studied variants are rs2231137 (c.34G>A) and rs2231142 (c.421C>A). The c.421C>A variant (rs2231142) is located in exon 5 and lies within the nucleotide binding domain of the transporter (Fohner et al., 2017). A decrease in BCRP expression is seen due to degradation of the variant protein in the endoplasmic reticulum. The c.421C>A variant affects the pharmacokinetics, response and toxicity of BCRP substrate substances, including chemotherapeutic agents (Stamp et al., 2010).

The accessibility of genotyping is subject to different factors, starting from the availability of such tests regionally to the local/national regulations governing their use. In certain countries like Switzerland, genotyping tests are covered by health insurance and are being more and more used to either screen the patients pre-emptively in an effort to reduce their risk of adverse effects or to identify the cause of the toxicity afterhand. This is in addition to the phenotyping tests currently available that help guide different pharmacotherapy with a narrow therapeutic index. There is ongoing debate regarding which screening test, if any, between the phenotyping and genotyping is superior depending on the molecule in question.

In summary, our patient had polymorphisms that put her at an increased risk of methotrexate toxicity. By identifying these genetic abnormalities prior to the first dose of methotrexate, it would have been possible to either to change the chemotherapy regimen or perform an unguided dose reduction of methotrexate in the absence of alternatives. Precision medicine, including pharmacogenomics, makes it possible to individualize the treatment of each patient to balance its therapeutic/toxic profile in the benefit of the patient. A prospective randomized study with methotrexate dose adjustments based on the patients’ polymorphisms would allow us to better anticipate the dose-dependent complications of methotrexate toxicity, and minimise the mortality-morbidity burden.

## Data Availability

The original contributions presented in the study are included in the article/supplementary materials, further inquiries can be directed to the corresponding author.
